# Effects of Organoboron Antifoulants on Oyster and Sea Urchin Embryo Development

**DOI:** 10.3390/ijms14010421

**Published:** 2012-12-24

**Authors:** Noritaka Tsunemasa, Ai Tsuboi, Hideo Okamura

**Affiliations:** 1 Environment Conservation Division, Environment Bureau, City of Hiroshima, 1-6-34 Naka-ku, Hiroshima 730-8586, Japan; 2 Lab Maritime Environment Management Graduate School of Maritime Science, Kobe University, 5-1-1 Fukaeminami, Higashinada-ku, Kobe 658-0022, Japan; E-Mails: aixxxu_uxxx@yahoo.co.jp (A.T.); okamurah@matitime.kobe-u.ac.jp (H.O.)

**Keywords:** bioassay, TPBP, TPBOA, alternative antifoulant, LC10, LC50

## Abstract

Prohibition of Ot (organotin) compounds was introduced in Japan in 1997 and worldwide from September 2008. This meant that the production of paints containing TBT compounds was stopped and alternatives to the available Ot antifoulants had to be developed. It has been claimed that the degradation by-products of these alternative antifoulants were less toxic than those of Ot compounds. Since the introduction of the alternative antifoulants, the accumulation of these compounds has been reported in many countries. However, the toxicity of these compounds was still largely unreported. In this research, the toxicity of the alternative Ot antifoulants TPBP (triphenylborane pyridine) and TPBOA (triphenylborane octadecylamine) and their degradation products on *Crassostea gigas* and *Hemicentrotus pulcherrimus* were tested. The results showed that toxic effects in *Crassostea gigas* was higher for each antifouling biocide than that in *Hemicentrotus pulcherrimus*. Also, while the toxicity of the Organoboron antifoulants and the Ots were the same, the former’s degradation products were much less harmful.

## 1. Introduction

Organotin compounds (Ots), used for many years as antifouling biocides on ships, marine structures, and fishing nets, became a problem because of their toxicity and accumulation characteristics.

The movement toward the global regulation of these compounds began in October 2001 when the International Maritime Organisation (IMO) adopted the International Convention on the Control of Harmful Antifouling Systems (AFS Convention), which prohibited the use of Ots as active ingredients in antifouling agents for marine vessels. This convention came into effect on 17 September, 2008.

Currently, Irgarol 1051^®^, Diuron, Sea-nine 211^®^, copper pyrithione, and other organic booster biocides are the major antifoulants used worldwide. In Tsunemasa and Okamura [1], a toxicity evaluation method on fertilized oyster egg was developed, and the toxicity of organotin alternative antifoulants (Irgarol 1051, Diuron and Sea-Nine 211) and organotin compounds (TBT and TPT) was evaluated. From these results, it was clear that Sea-Nine 211 was as toxic as organotin compounds to fertilized oyster eggs.

Some organoboron antifoulants such as TPBP and TPBOA are only used in Japan and some other Asian countries, but there is little data available on their impact, bioaccumulation, and environmental toxicity. Only the research on *Artemia salina* and *Skeletonema costatum* [2] and that on *Anthocidaris crassispina* [3] have looked at organoboron antifoulants.

The first biological examination of sea urchins was started by Kobayashi *et al.* [4], followed by Loenning and Hagstroem [5], Dinnel *et al.* [6], Pagano *et al.* [7], and Beiras *et al.* [8]. The government agencies Environment Canada [9], US EPA [10], ASTM [11] and APHA [12] conducted their own research based on earlier test methods used on fertilized sea urchin eggs. In the research for this report, the revised Kobayashi method [13] was used to determine the effects on embryonic development of sea urchins.

Oysters and sea urchins were used in this research due to their high sensitivity to chemical compounds [14–16], as well as the fact that oysters and sea urchins can be found globally.

First, the rate of fertilization, development after fertilization, rate of deformity in the embryos, and number of underdeveloped embryos were measured. Then the effects of alternative Organoboron antifoulants on *Crassostea gigas* and *Hemicentrotus pulcherrimus* embryos were evaluated. The toxicity of *Crassostea gigas* and *Hemicentrotus pulcherrimus* were compared.

## 2. Results

**Figure 1:**
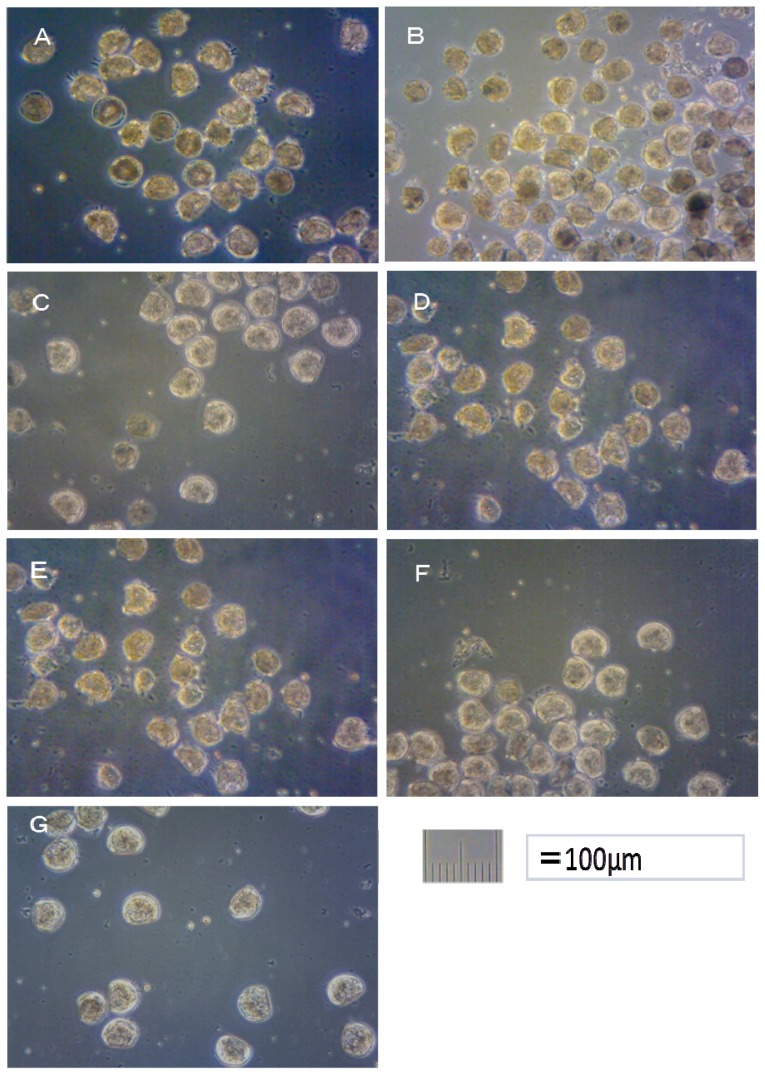
Effects of TPBP and TPBOA on *Crassostea gigas* embryo development after 24 h. (**A**) TPBP at 10 μg/L; (**B**) TPBP at 1 μg/L; (**C**) TPBP at 0.1 μg/L; (**D**) TPBOA at 10 μg/L; (**E**) TPBOA at 1 μg/L; (**F**) TPBOA at 0.1 μg/L; (**G**) Control.

**Figure 2:**
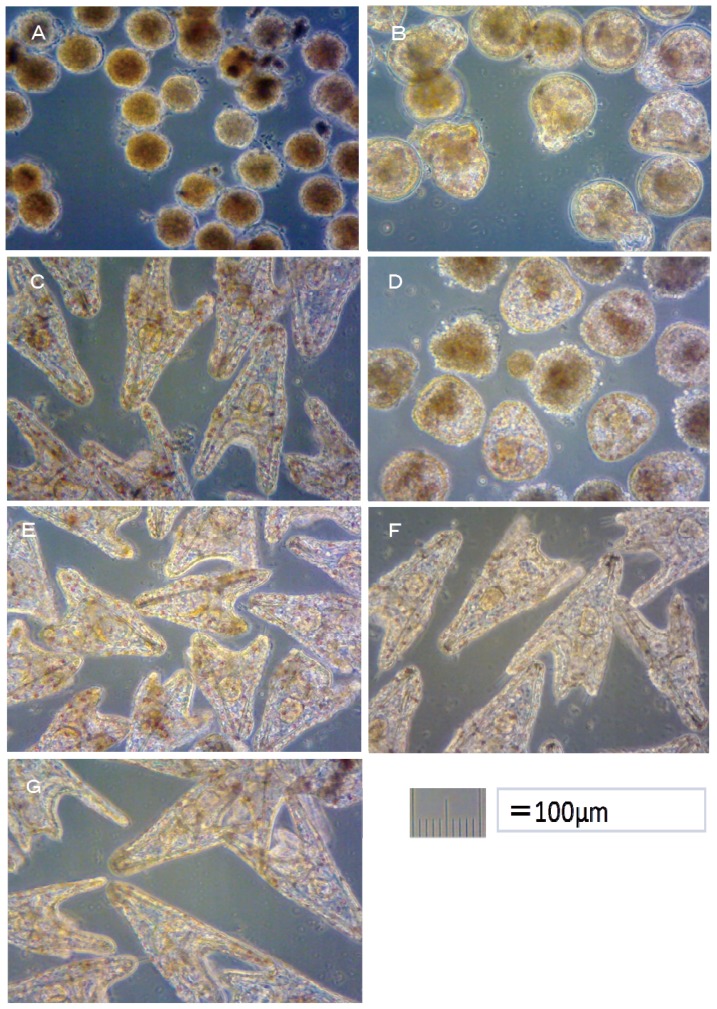
Effects of TPBP and TPBOA on *Hemicentrotus pulcherrimus* embryo development after 48 h. (**A**) TPBP at 100 μg/L; (**B**) TPBP at 50 μg/L; (**C**) TPBP at 20 μg/L; (**D**) TPBOA at 100 μg/L; (**E**) TPBOA at 50 μg/L; (**F**) TPBOA at 20 μg/L; (**G**) Control.

The alternative Ot antifoulants (TPBP and TPBOA) and the degradation products from TPBP and TPBOA (e.g., DPB, MPB, biphenyl, phenol, pyridine, benzene and boric acid), at concentrations ranging from 0.1 to 1000 μg/L, were used in the toxicity test on the fertilized *Crassostea gigas* and *Hemicentrotus pulcherrimus* eggs. The photograph of *Crassostea gigas* embryo development after 24 h at 25 °C is shown in Figure 1. Using these images, the toxicity of antifouling biocides was evaluated by examining cell division at 2 h after fertilization and checking embryology (*i.e.*, for D-shaped embryos) at 24 h after fertilization. The photograph of *Hemicentrotus pulcherrimus* embryo development after 48 h at 20 °C is shown in Figure 2. Using these images, the toxicity of antifouling biocides was evaluated by examining cell division at 10 h after fertilization and checking embryology (*i.e.*, for two-armed echinopluteus embryos) at 48 h after fertilization. Controls after 24 h of *Crassostea gigas* and controls after 48 h of *Hemicentrotus pulcherrimus* were normal. Controls after 24 h of *Crassostea gigas* and controls after 48 h of *Hemicentrotus pulcherrimus* are shown in Figures 1G and 2G, respectively.

### 2.1. Effects of Organoboron Antifoulants on *Crassostea gigas* Embryo

#### 2.1.1. TPBP and TPBOA

**Figure 3:**
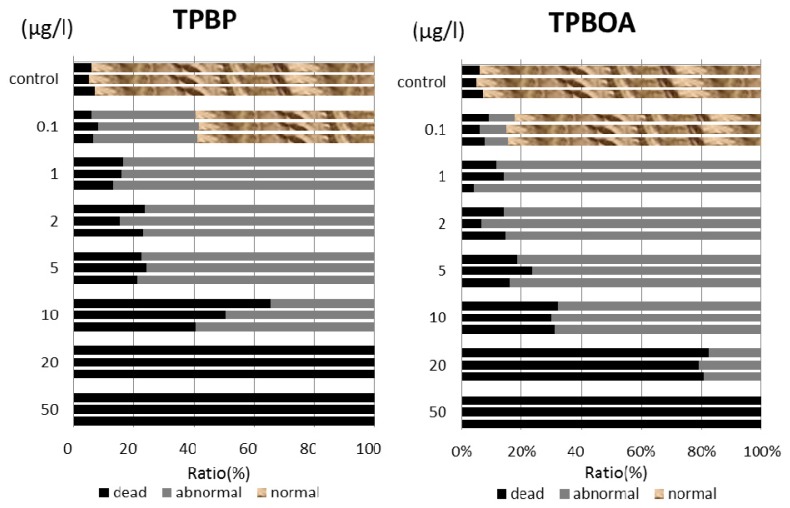
Survival and protruding-mantle deformity rates on *Crassostea gigas* embryo after a period of 24 h.

In this report, the survival rate of fertilized eggs and the occurrence of deformity in D-shaped embryos were investigated. Survival rate and deformity rates of *Crassostea gigas* embryo after a period of 24 h are shown in Figure 3. In the report by His *et al.* [17], four types of deformity were shown: convex hinge, indented shell margin, incomplete shell, and protruding mantle. In our experiments, only protruding-mantle deformities were observed.

In the case of TPBP, all of *Crassostea gigas* eggs in the 100 μg/L treatment died after 2 h, before any cell division could take place. Approximately 25% of *Crassostea gigas* eggs in the 10 μg/L treatment died before any cell division had occurred. Approximately 5% of the embryos which survived showed signs of deformity or delayed development. Approximately 10% of *Crassostea gigas* eggs in the 1 μg/L treatment died before any cell division. A small percentage of the embryos which survived showed signs of deformity or delayed development.

Approximately 50% of *Crassostea gigas* eggs in the 10 μg/L treatment died after 24 h. Half of the eggs showed no signs of cell division, but in the case of the other half, cell division had occurred before death. All of the surviving embryos, which became D-shaped embryos, developed protruding-mantle deformity (Figure 1A). Approximately 85% of *Crassostea gigas* eggs in the 1 μg/L treatment survived. Half of the embryos became D-shaped embryos but half showed signs of delayed development. Almost all of the D-shaped embryos developed protruding mantle deformity (Figure 1B). Almost all of *Crassostea gigas* eggs in the 0.1 μg/L treatment survived. Approximately 70% of embryos became a D-shaped embryo, while the other 30% of the embryos showed signs of delayed development. Most D-shaped embryos were normal, but slightly less than 10% developed protruding-mantle deformity (Figure 1C).

In the TPBOA samples, approximately 85% of *Crassostea gigas* eggs in the 100 μg/L treatment died after 2 h, before any cell division could take place. Slightly less than 20% of *Crassostea gigas* eggs in the 10 μg/L treatment died before any cell division took place. A small percentage of the embryos which survived showed signs of deformity or delayed development. Slightly less than 10% of *Crassostea gigas* eggs in the 1 μg/L treatment died before any cell division had taken place. A small percentage of the embryos which survived showed signs of deformity or delayed development.

Slightly more than 30% of *Crassostea gigas* eggs in the 10 μg/L treatment died after 24 h. Most eggs showed no signs of cell division, but in a few cases, cell division had occurred before death. Half of the embryos became D-shaped embryos and half showed signs of delayed development. Almost all of the D-shaped embryos developed protruding-mantle deformity (Figure 1D). Slightly less than 10% of *Crassostea gigas* eggs in the 1 μg/L treatment died. Approximately 10% of the embryos which survived showed signs of delayed development, the other embryos became D-shaped embryos. Almost all of the D-shaped embryos developed protruding-mantle deformity (Figure 1E). Most of the eggs in the 0.1 μg/L treatment survived. All the surviving embryos became D-shaped embryos. Most D-shaped embryos were normal with slightly less than 10% of the D-shaped embryos developed protruding-mantle deformity (Figure 1F).

#### 2.1.2. Degradation Products from TPBP and TPBOA

*Crassostea gigas* eggs had developed favorably after 2 and 24 h, and no evidence of any influence on the development of the embryos was found, even at the maximum concentration of degradation products (DPB, MPB, Biphenyl, Phenol, Pyridine, Benzene, Boric acid) studied (1000 μg/L).

#### 2.1.3. LC_10_ and LC_50_ Values

The LC_10_ and LC_50_ values of each compound were calculated from the survival rate of the fertilized *Crassostea gigas* eggs after the exposure times of 2 and 24 h using the Ecotox-Statics software package. The results are listed in Table 1. It can be seen that the toxicity of the degradation products was not influential on *Crassostea gigas* embryos at high concentrations (1000 μg/L). In the case of the other compounds, at 2 h the LC_10_ and LC_50_ values of TPBP and TPBOA were 1.1 (3.4) and 2.7 (5.1) μg/L (nM), 7.5 (23) and 23 (44) μg/L (nM), respectively. At 24 h, the LC_10_ and LC_50_ values of these compounds were 0.58 (1.8) and 2.2 (4.2) μg/L (nM), 6.3 (20) and 10 (19) μg/L (nM), respectively.

### 2.2. Effects of Organoboron Antifoulants on *Hemicentrotus pulcherrimus* Embryos

#### 2.2.1. TPBP and TPBOA

**Figure 4:**
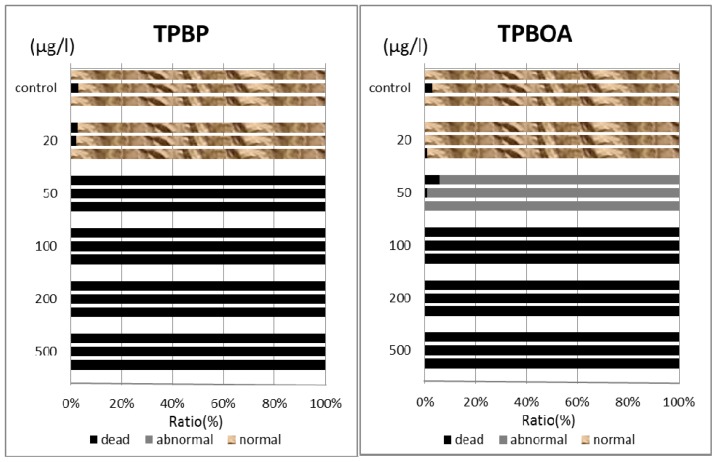
Survival and unequal length of postoral arms deformity rates on *Hemicentrotus pulcherrimus* embryo after a period of 48 h.

**Figure 5:**
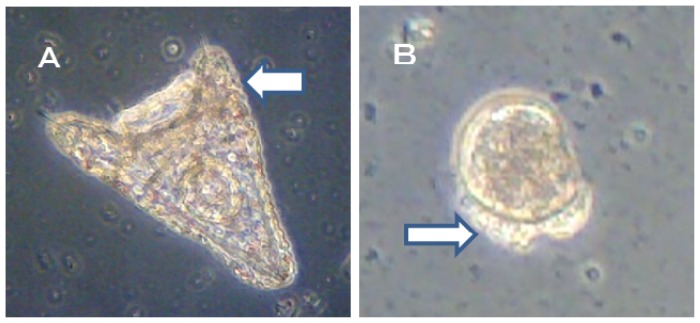
Deformity embryo (**A**) *Hemicentrotus pulcherrimus* (**B**) *Crassostea gigas.*

In this report, the survival rate of fertilized eggs and the occurrence of deformity in *Hemicentrotus pulcherrimus* embryos were investigated. Survival and deformity rates of the *Hemicentrotus puldherrimus* embryo after a period of 48 h are shown in Figure 4. In the report by His *et al.* [18], six types of deformity were shown: unequal length of postoral arms, twisted right oral rod, parts of right body rod missing, left body rod doubled, additional crossbarred body rod and apically “crossed” body rod. In our experiments, slight unequal length of postoral arms deformity was observed. Deformity embryos are shown in Figure 5.

In the case of TPBP, all of *Hemicentrotus puldherrimus* eggs in the 100 μg/L treatment died after 10 h before any cell division could take place. Approximately 10% of *Hemicentrotus puldherrimus* eggs in the 50 μg/L treatment died before any cell division had occurred. In the eggs which survived, there were no signs of deformity or delayed development. Approximately 5% of *Hemicentrotus puldherrimus* eggs in the 20 μg/L treatment died before any cell division. The surviving embryos showed no signs of deformity or delayed development.

All of *Hemicentrotus puldherrimus* eggs in the 100 μg/L treatment died after 48 h before any cell division could take place (Figure 2A). Almost all of *Hemicentrotus puldherrimus* eggs in the 50 μg/L treatment died. Except for the eggs which died before any cell division took place, almost all of the embryos reached the blastula stage. A few of them developed signs of reaching the gastrula stage (Figure 2B). Almost all of *Hemicentrotus puldherrimus* eggs in the 20 μg/L treatment survived. All surviving embryos became normal two-armed echinopluteus embryos (Figure 2C).

In the TPBOA samples, all of *Hemicentrotus puldherrimus* eggs in the 500 μg/L treatment died after 10 h before any cell division could take place. Approximately 10% of *Hemicentrotus puldherrimus* eggs in the 200 μg/L treatment died before any cell division took place. In the embryos which survived, there were no signs of deformity or delayed development. Approximately 5% of *Hemicentrotus puldherrimus* eggs in the 100 μg/L treatment died. No cell division had taken place. There were no signs of deformity or delayed development in the surviving embryos.

All of *Hemicentrotus puldherrimus* embryos in the 100 μg/L treatment died after 48 h. Almost all of them reached the gastrula stage (Figure 2D). Almost all of the embryos in the 50 μg/L treatment survived. All surviving embryos became two-armed echinopluteus embryos. All of the echinopluteus developed short arms (Figure 2E). Almost all of the embryos in the 20 μg/L treatment survived. All surviving embryos became normal two-armed echinopluteus embryos (Figure 2F).

#### 2.2.2. Degradation Products from TPBP and TPBOA

*Hemicentrotus puldherrimus* eggs had developed favorably after 10 and 48 h, and no evidence of any influence on the development of the embryos was found, even at the maximum concentration of degradation products (DPB, MPB) studied (1000 μg/L).

#### 2.2.3. LC_10_ and LC_50_ Values

The LC_10_ and LC_50_ values of each compound were calculated from the survival rate of the fertilized *Hemicentrotus pulcherrimus* eggs after exposure times of 10 and 48 h using the Ecotox-Statics software package. The results are listed in Table 2. It can be seen that the toxicity of the degradation products was not influential on *Hemicentrotus pulcherrimus* embryos at high concentrations (1000 μg/L). In the case of the other compounds, at 10 h the LC_10_ and LC_50_ values of TPBP and TPBOA were 22 (68) and 130 (250) μg/L (nM), 73 (230) and 290 (550) μg/L (nM), respectively. At 48 h, the LC_10_ and LC_50_ values of these compounds were 6.4 (20) and 30 (57) μg/L (nM), 31 (96) and 73 (140) μg/L (nM), respectively.

## 3. Discussion

At 24 h, the LC_50_ values of TBT, triphenyltin (TPT), Sea-Nine 211, Diuron and Irgarol 1051 in *Crassostea gigas* embryos were 3.9, 3.7, 17, >1000, and >1000 μg/L, respectively [1]. TPBP, TBT and TPT had almost the same toxicity in *Crassostea gigas* embryos. TPBOA and Sea-Nine 211 also had similar levels of toxicity as one another in *Crassostea gigas* embryos.

The results of our laboratory tests showed that organoboron antifoulants are as toxic as Ots to the oysters and sea urchins. According to the results of this research and previous research [1], the effects of antifouling biocides on oysters are shown as follows:

TBT=TPT=TPBP>Sea-Nine 211>TPBOA>>Diuron=Irgarol 1051

Previous toxicity data of Ot alternative antifoulants on *Hemicentrotus pulcherrimus* could not be found, so the results of this research were compared with previously reported no observed-effect concentration (NOEC) data on Zinc pyrithione, Chlorothalonil and Sea-Nine 211 from the sea urchin (*Paracentrotus lividus*). The values for *Paracentrotus lividus* were 11 nM (3.49 μg/L), 15 nM (3.98 μg/L) and 23 nM (6.49 μg/L), respectively [19]. Therefore, the toxicity of TPBP in the *Hemicentrotus pulcherrimus* (in this case, the LC_10_ value at 48 h was used) was almost the same level as the *Paracentrotus lividus*. However, that of TPBOA in the *Hemicentrotus pulcherrimus* was a little lower than the *Paracentrotus lividus*.

According to the results of this research and previous researches [20], the effects of antifouling biocides on sea urchins are shown as follows:

TBT>TPBP=Sea-Nine 211>TPBOA>>Irgarol 1051

The toxicity test in the *Anthocidaris crassispina* showed that the toxicity of TPBP and Sea-Nine 211 was high and that of Irgarol 1051 and Diuron was low [3].

These results showed almost the same pattern. However, the toxicity of each antifouling biocide showed that the toxicity in oysters was higher than that in sea urchins. At first, it was thought that this tendency was due to the presence of a fertilization membrane in sea urchins which oysters do not have. However, the fertilization membrane is less dense than the cell membrane, so it is difficult to believe that the fertilization membrane can prevent any materials passing through that the cell membrane could not. Therefore, the difference of the toxicity in oysters and sea urchins could not be explained from this study’s results.

## 4. Experimental Section

### 4.1. Reagents and Materials

**Figure 6:**
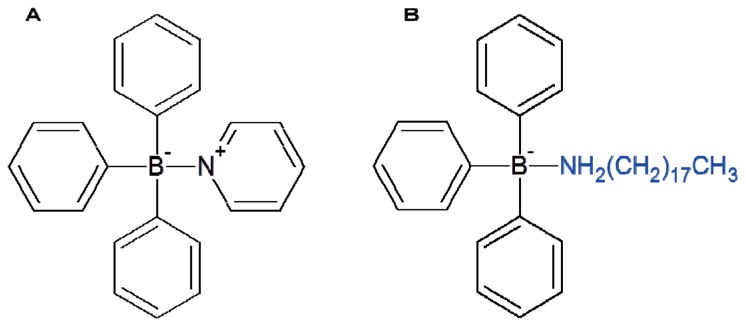
Chemical structures of triphenylborane compounds tested. (**A**) TPBP (**B**) TPBOA.

Triphenylborane pyridine (TPBP) and triphenylborane octadecylamine (TPBOA) were used as the antifoulants in this study. The chemical structures of TPBP and TPBOA are shown in Figure 6. Diphenylborane hydroxide (DPB), phenylborane dihydroxide (MPB), biphenyl, pyridine, phenol, benzene, and boric acid were used as the degradation products from TPBP or TPBOA [2,21]. The TPBP, DPB, and MPB were donated by Hokko Chemical Industry (Tokyo, Japan). The TPBOA was donated by Benny-Toyama (Osaka, Japan). The phenol, benzene, biphenyl (pesticide grade), and pyridine (spectroscopy grade) were purchased from Wako Pure Chemical Industries (Osaka, Japan). The boric acid was obtained from Nakarai Chemical K.K. (Ibaraki, Japan). Dilute stock solutions (1000 mg/L) were prepared by dissolving the standard materials in dimethyl sulfoxide (DMSO). The standard solutions (0.1, 0.5, 1.0, 2.0, 5.0, 10, 20, 50, 100, 200, 500, 1000 μg/L) were formed by diluting these solutions with artificial seawater which was prepared by diluting Daigo’s artificial seawater SP purchased from Nihon Seiyaku Kogyo (Niigata, Japan). The dimethyl sulfoxide (for biochemistry) and 10% formalin solution (for tissue fixation) were purchased from Wako Pure Chemical Industries (Osaka, Japan). An alkaline formalin solution was prepared by further diluting these solutions with the artificial seawater stock. The oysters were gathered from the breakwater in Itsukaichi Nishi Ward, Hiroshima, Japan. Professor Kenji Torigoe, who is affiliated with the Department of Education at Hiroshima University, identified the oysters used in the experiments. All the oysters used were *Crassostrea gigas*. The sea urchins were purchased from Taguchi Educational Laboratory. All the sea urchins used were *Hemicentrotus pulcherrimus* from the Miura Peninsula.

### 4.2. Equipment

An Olympus CK40 biological microscope with a magnification of 100× was used to photograph the oyster and sea urchin eggs, and the Motic Images Plus 2.2S image editing software package was used to count the number of oyster and sea urchin eggs.

### 4.3. Oyster Toxicity Tests

After some initial trial and error tests, it was decided to use the procedure performed at the Fisheries Experimental Station in Hiroshima Prefecture, which simulates the conditions in a nursery. The artificial seawater was bubbled for 1–2 h to oxygenate the solution. The oyster’s shell was cut open with a scalpel, and the sex organs were removed. Then a slight incision was made in the sex organs and a sample collected; the sample was observed under the microscope; and the sex of the oyster was determined. The scalpel used in the experiment was washed under running water each time a sample was removed. The male oysters were placed in a laboratory dish and kept in a refrigerator. This cold storage preservation stage was a new stage introduced in the experiments. Because of this, it was possible to use these oysters in the experiments for >1 day. As in many cases, it was important to use samples freely.

In the case of female oysters, beakers filled with artificial seawater were covered with a fine net; the sex organs were placed on top of the beaker; and then the sex organs were dissected. The eggs were collected in the beakers and then washed in artificial seawater. The eggs were washed with artificial seawater several times to separate the mature eggs from the immature eggs. Only the mature eggs that settled at the bottom of the beaker were used, unlike the reports by His *et al.* [17,18], who used the ASTM process to select the eggs [22]. A volume of 10 mL of standard solution was placed into a six-hole microplate. The controls were adding DMSO to the artificial seawater at 0.1%. Three wells with the same concentration were prepared. Approximately 200 mature eggs were added to each well along with a volume of 25 μL artificial seawater. The sperm, which was preserved in a refrigerator, was diluted with artificial seawater 1000 times; a volume of 100 μL of artificial seawater was added to each well; and then the well was used to fertilize the samples. This marked the beginning of the toxicity tests. A constant temperature tank, maintained at 25 °C, was used for six-well microplates during cultivation. Each well was observed under the microscope at 2 and again at 24 h. The development stages of 200 oyster eggs or embryos (normal and abnormal) were identified and photographed. After the experiments were concluded, the 10% lethal concentration (LC_10_) and the 50% lethal concentration (LC_50_) values were calculated using the Ecotox-Statics software program developed by the Japanese Society of Environmental Toxicology.

### 4.4. Sea Urchin Toxicity Tests

The sea urchin’s body fluid was drained after removing a part of their mouth. The gonad’s color was then confirmed. If the color was orange, it was identified as an ovary. If the color was light yellow or white, it indicated a testis. And then, they were injected with 1 mL of 0.5 M KCL in a part of the mouth. The male sea urchins were placed on their backs in a laboratory dish and kept in a refrigerator and a high density of sperm was collected. In the case of female sea urchins, a volume of 100 mL of the Erlenmeyer flask was filled with artificial seawater, the sea urchins were placed on their backs on top of the flask. The eggs were collected in the flask and then washed in artificial seawater several times to separate the mature eggs from immature eggs. A constant temperature tank, maintained at 20 °C, was used for six-well microplates during cultivation. Each well was observed under the microscope at 10 and again at 48 h. The development stages of 200 sea urchin eggs or embryos (normal and abnormal) were identified and photographed. The other operations were performed the same as the oysters toxicity test.

## 5. Conclusions

The effect before degradation of TPBP on oyster and sea urchin eggs and embryos was high but its degradation products showed no evidence of any influence.

24 h-LC_50_ of TPBP on oyster embryos had almost the same level of toxicity as that of TBT and TPT.

48 h-LC_50_ of TPBP on sea urchin embryos had almost the same level of toxicity as Sea-Nine 211 but was lower than TBT.

The effect of TPBOA on oyster and sea urchin eggs and embryos was lower than TPBP.

The toxicity of TPBP and TPBOA caused protruding-mantle deformity in the oyster embryos, and caused unequal length of postoral arms deformity in the sea urchin embryos.

## Figures and Tables

**Table 1:** Lethal concentrations of antifouling compounds to oyster *Crassostea gigas* embryos.

	2 h		24 h	

	LC10	LC50	LC10	LC50
TPBP	1.1 (1.0–1.1)	7.5 (6.7–8.5)	0.58 (0.55–0.60)	6.3 (5.4–7.4)
TPBOA	2.7 (2.6–2.8)	23 (20–26)	2.2 (2.1–2.8)	10 (9.5–12)
DPB	>1000	>1000	>1000	>1000
MPB	>1000	>1000	>1000	>1000
Biphenyl	>1000	>1000	>1000	>1000
Phenol	>1000	>1000	>1000	>1000
Pyridine	>1000	>1000	>1000	>1000
Benzene	>1000	>1000	>1000	>1000
Boric acid	>1000	>1000	>1000	>1000

LC50: 50% lethal concentration (μg/mL); LC10: 10% lethal concentration (μg/mL); () 95% confidence interval.

**Table 2:** Lethal concentrations of antifouling compounds to sea urchin *Hemicentrotus pulcherrimus* embryos.

	2 h		48 h	

	LC10	LC50	LC10	LC50
TPBP	22 (21–23)	73 (67–80)	6.4 (5.7–6.6)	31 (27–36)
TPBOA	130 (120–130)	290 (270–320)	30 (28–32)	73 (68–79)
DPB	>1000	>1000	>1000	>1000
MPB	>1000	>1000	>1000	>1000

LC50: 50% lethal concentration (μg/mL); LC10: 10% lethal concentration (μg/mL); () 95% confidence interval.
